# Ultrasound Guidance Superiority in Pediatric Sialorrhea Treatment With Intraglandular Botulinum Toxin Application: A Four-Year Retrospective Study

**DOI:** 10.7759/cureus.45359

**Published:** 2023-09-16

**Authors:** Mafalda Pires, Joana Saldanha, Sandra Claro

**Affiliations:** 1 Physical Medicine and Rehabilitation (PM&R) Department (Paediatrics Area), Centro Hospitalar Universitário de Lisboa Central, Lisbon, PRT; 2 Physical Medicine and Rehabilitation Department, Centro Hospitalar Baixo Vouga, Aveiro, PRT

**Keywords:** botulinum neurotoxin type-a, botulinum toxin injection, ultrasonography, pediatric, sialorrhea

## Abstract

Introduction

The management of sialorrhea in children with multiple disabilities is extremely important not only for aesthetic/psychosocial reasons but also for functional and clinical ones. There are several recommended management methods with strong evidence of the effectiveness of intraglandular application of botulinum toxin A.

Materials and methods

In this four-year retrospective report, we compare two populations who received intraglandular type A botulinum toxin injections in the pediatric unit of the Physical Medicine and Rehabilitation (PM&R) Department at a central hospital. The injections were administered using either ultrasound guidance (US) or anatomical landmarks.

Results

Out of a total of 29 patients with neurological conditions, 16 met the eligibility criteria for this study. The study group comprised seven females (44%) and nine males (56%), with a median age of 9 years. The average pre-procedure sialorrhea staging was four. A total of 23 procedures were performed, with 16 conducted under ultrasound guidance (US) and seven via anatomical landmarks (non-US). In the US group, a statistically significant difference in sialorrhea staging was observed at one and three months post-procedure (p<0.05), but not at six months post-procedure. Conversely, no statistically significant difference in sialorrhea staging was found at any time point in the non-US group. The comparison between the two groups supports the use of ultrasound guidance, showing superior outcomes at one and three months post-procedure (p<0.05).

Conclusion

The results of this study align with global trends seen in medical publications and guidelines advocating for the use of ultrasound in this procedure. Future prospective and larger-scale studies are essential to validate these findings.

## Introduction

Sialorrhea (drooling) is defined as an excessive accumulation of saliva in the oral cavity combined with unintentional saliva loss [1]. This condition may have multiple etiologies.

In the context of pediatric rehabilitation, sialorrhea can be more frequently determined by (a) loss/increase/anatomical distortion of facial, oral, and upper digestive tract structure (e.g., cleft lip, retrognathism, macroglossia, tonsillar hypertrophy, rhinopharyngeal polyps, esophageal stenosis, diverticula); (b) loss or alteration of central/peripheral neurological or muscular function of the lips, tongue, facial, and pharyngolaryngeal muscle control (e.g., cerebral palsy, central facial paresis, peripheral facial palsy, myasthenia gravis, Moebius syndrome, and Guillain-Barré syndrome) [2].

Sialorrhea causes health and psychological problems in these patients, leading to stress and social isolation for the family and the patient. Children who suffer from sialorrhea may have damage and dermatitis of the skin around the mouth, electrolyte disorders, and even dehydration [2].

As a result, intervention for this symptom/sign is important, and its approach should be based on a progressively invasive medical intervention [3].

Each patient evaluation should include a qualitative and quantitative drooling assessment, which is of utmost importance in evaluating the outcomes of medical interventions.

Scales typically used include the drooling severity and frequency scale or Stonell-Greenberg scale; the number of bibs/tissues used to contain/clean saliva; and patient global impression of change [4].

In the context of sialorrhea treatment, the initial approach in the majority of cases involves nonpharmacological and conservative interventions. These typically encompass behavioral modifications and oral-motor exercises administered by speech and language therapists [4].

Access to first-line medications like glycopyrronium bromide oral suspension and transdermal scopolamine [5] is restricted in Portugal, primarily because of economic constraints and limited availability in both hospitals and community pharmacies.

The utilization of alternative anticholinergic medications, such as sublingual atropine and inhaled ipratropium bromide, is primarily limited due to frequent iatrogenic side effects, including severe xerostomia, urinary retention, and constipation. These side effects can be profoundly distressing for patients with severe neurological conditions. Moreover, the efficacy of these alternatives hinges on patient compliance [6].

The intraglandular injection of botulinum toxin type A (BTX-A) appears as an option in situations where non-pharmacological treatment is not feasible or effective, and systemic medication is not tolerated [6,7]. 

A 2012 Cochrane review reports some evidence for the short-term benefits of both medication and BTX-A; however, no conclusions were reached on the efficacy and safety of either BTX-A or glycopyrrolate in the treatment of drooling in children with cerebral palsy [8].

At our hospital, this treatment option has been available for approximately 10 years, exclusively using onabotulinum toxin A. For the initial six years, the injections were performed solely based on anatomical landmarks. However, since 2019, we have incorporated ultrasound-guided BTX-A intraglandular application into our practice, employing a portable multifrequency ultrasound device (GE Healthcare Vscan Air Ⓡ).

A physical medicine and rehabilitation (PM&R) team dedicated to the treatment of sialorrhea assesses the child using a clinical questionnaire and evaluation tool, which includes:

a) Anamnesis ((1. baseline pathology; 2. characterization of sialorrhea with the application of Thomas-Stonell and Greenberg/drooling severity and frequency Scale; 3. oral habits/eating habits/oral hygiene habits; 4. medical history (extrathoracic respiratory tract disorders; neuromuscular plate diseases/second motor neuron/myopathies) including clinical contraindication for the use of anticholinergic drugs; 5. surgical history (otorhinolaryngology and maxillofacial surgery); 6. allergies (drugs and others) including hypersensitivity to BTX-A or any of its components; 7. usual medication including current use of systemic anticholinergic agents; 8. prior/current sialorrhea treatment and results obtained; and 9. history of past BTX-A injection (date and results)).

b) Objective examination (1. ability to comply with oral-motor commands; 2. resting posture of the lower jaw and cranio-cervical alignment; 3. abnormal movements of the lower jaw (trismus/ bruxism); and 4. dentition and circumoral skin condition).

The injected botulinum toxin is onabotulinumtoxinA 100U (diluted in 1 ml of saline). Administrations are performed following the standards of aseptic technique. The posology is based on the patient's weight, according to Lungren et al*.* [9]: 15 units/gland if weighing <15 kg; 20 units/gland if weighing 15-25 kg; and 25 units/gland if weighing >25 kg.

Patients’ evaluation and BTX-A posology follow the same protocol for both types of procedures:

(a) For the anatomic-landmark-based procedure [10], the administration is divided between the parotid and submandibular glands as follows: two points in each parotid gland (1 cm anterior to the tragus and in the transition region between the parotid and submandibular glands) and one point in each submandibular gland (1 cm anterior to the mandibular angle).

b) For the ultrasound-guided procedure [11], we employed a high-frequency US linear probe with a frequency exceeding 10 MHz to visualize both the salivary glands and the adjacent neurovascular structures:

1) For the evaluation of the parotid gland, the longitudinal axis of the device is placed transverse to the vertical ramus of the mandible (the posterior maxillary vein and the external carotid must be identified).

2) For the evaluation of the submandibular gland, the longitudinal axis of the probe is placed relative to the horizontal ramus of the inferior maxillary (the inferior maxillary artery must be identified).

Following the administration of BTX-A, we conduct remote consultations with parents/caregivers via telephone to assess the outcomes.

This follow-up is made at the end of the first-, third-, and sixth-month post-procedure, with drooling severity and frequency scale evaluation. The aim of the current study was to retrospectively compare the effectiveness of BTX-A injection on sialorrhea treatment when guided by ultrasound versus anatomical landmarks.

## Materials and methods

This retrospective study assessed medical records of BTX-A sialorrhea treatments at a tertiary pediatric hospital's PM&R department between 2019 and 2022. We gathered demographic data, including gender, baseline disease, and age at the time of treatment. Additionally, we recorded procedure details such as the use of US guidance or anatomical landmarks. For each procedure, we collected sialorrhea staging at four different time points: before treatment and at one, three, and six months after treatment.

Inclusion criteria: Consultation in the PM&R department; age from three years to 19 years; sialorrhea in the context of neurologic disease (congenital/genetic or acquired); pretreatment sialorrhea staged 3 or higher on the Thomas-Stonell and Greenberg/drooling severity and frequency scale (Table 1); recording of the pretreatment staging and at least one reevaluation after the procedure; cases in which BTX-A was injected in four salivary glands (two parotid and two submandibular) at each treatment.

**Table 1:** Thomas-Stonell and Greenberg/Sialorrhea severity and frequency scale. *For ease of data evaluation, we present the staging in Arabic numerals instead of Roman numerals.

Severity (S)	Frequency (F)	Staging (St)*
1- Dry: no sialorrhea	1- No sialorrhea	1: S1; F1
2- Wet: only wet lips	2- Occasional	2: S2, S3; F2
3- Moderate: lips and neck	3- Frequent	3: S3; F3, F4
4- Severe: clothes	4- Constant	4: S4, S5; F2, F3, F4
5- Profuse: clothes, hands, objects		

Exclusion criteria: Noncompliance with the inclusion criteria; sialorrhea staging using other scales; BTX-A dosage out of the clinical evaluation tool (see the Introduction).

The statistical analysis was conducted using Microsoft Excel® and Statistical Product and Service Solutions (SPSS) (IBM SPSS Statistics, v29.0, for Windows, Armonk, NY). The distribution of data was assessed with histograms and Q-Q plots and nonparametric tests were used to compare non-Gaussian distributed data. Specifically, we used the Wilcoxon signed-rank test for one-population evaluation and the Mann-Whitney U test when comparing two populations. Data are reported as medians (Mdn), (interquartile range), or proportions (%). Statistical significance was set at a threshold of p<0.050.

## Results

Out of a total of 29 identified patients, 16 were included in the study. Among them, there were seven females (44%) and nine males (56%), with ages ranging from 5 to 19 years at the time of the procedure. The median age was nine years. All included patients had a neurological condition (Table 2).

**Table 2:** Characteristics of patients admitted to the study. M=male; F=female

	SEX	AGE AT THE DAY OF INFILTRATION (years)	NUMBER OF REPETITIONS OF THE PROCEDURE	DIAGNOSIS
1	M	7	1	Cerebral palsy
2	M	16	1	Cerebral palsy
3	F	14	1	Congenital cytomegalovirus
4	F	10	1	Rett syndrome
5	F	9	1	Streptococcus pneumoniae meningitis + sepsis
6	F	9	1	Infantile childhood epileptic encephalopathy type 7 AD with a gene mutation on Kcnq2
7	M	8	1	Subdural and posterior subarachnoid and extracranial hemorrhage
8	M	8	4	Cerebral palsy
9	F	5	1	Pitt-Hopkins syndrome
10	M	9	1	Polymicrogyria
11	F	9	2	Cerebral palsy
12	M	13	1	Cerebral palsy
13	M	16	2	Variant SHROOM 4 OMIM Disease #300434 Stocco Dos Santos X-linked
14	M	19	1	Cerebral palsy
15	F	6	3	Cerebral palsy
16	M	13	1	Pallister Killian syndrome

A total of 23 procedures were performed, 16 were guided by ultrasound (US group), and seven were performed via anatomical landmarks (non-US group).

The separate evaluation of the two groups returned the data presented in Tables 3-4.

**Table 3:** Patient characteristics and results of the ultrasound-guided procedure (US group). M=male; F=female; St=staging

	PATIENT	SEX	PRE-TOXIN SIALORRHEA STAGING (STONNEL GREENBERG SCALE)		ONE-MONTH POST-TOXIN SIALORRHEA STAGING		THREE-MONTH POST-TOXIN SIALORRHEA STAGING		SIX-MONTH POST-TOXIN SIALORRHEA STAGING	
			St		St		St		St	
	1	M	4		4		4		4	
	2	M	4		1		1			
	3	F	4		3		4			
	4	F	4		2		2		2	
	5	F	4		2		2			
	6	F	4							
	7	M	3		3		3			
	8	M	4				3			
			4		4					
			3				2		2	
			3				2		2	
	9	F	3		3				4	
	10	M	3		3		3		3	
	11	F	4		2		2			
			4		2		4			
	12	M	4		4					
MEDIAN			4		3		2.5		2.5	

**Table 4:** Patient characteristics and results of the anatomical references-guided procedure (non-US group). M=male; F=female; St=staging

	PATIENT	SEX	PRE-TOXIN SIALORRHEA STAGING (STONNEL GREENBERG SCALE)		ONE-MONTH POST-TOXIN SIALORRHEA STAGING		THREE-MONTH POST-TOXIN SIALORRHEA STAGING		SIX-MONTH POST-TOXIN SIALORRHEA STAGING	
			St		St		St		St	
	13	M	4		4		4			
			4		3		3		4	
	14	M	4		4		4			
	15	F	4		4		4			
			4		4		4			
			4		4		4		4	
	16	M	4		4				4	
MEDIAN			4		4		4		4	

Both groups had a non-normal distribution.

In the US group, the results of a Wilcoxon Signed-Ranks test revealed a statistically significant difference in sialorrhea staging at both one month (Mdn=3; W=-2.264; p<0.024) and three months (Mdn=2.5; W=-2.401; p<0.016) following the procedure, with the most pronounced improvement observed at the three-month mark after infiltration.

No significant difference was found at six months after the procedure. Within the non-US group, no improvement was observed across any of the assessment time points.

The comparison of the two groups with the Mann-Whitney U test supports the use of US on salivary gland BTX-A infiltration, revealing evidence of superior outcomes at one month (U=10.00; Mdn=3; (2-4); p<0.008) and three months after the procedure (U=8.50; Mdn=3; (2-4); p<0.010).

## Discussion

The analysis of the results shows that the size of our sample resembles the size of other cohorts studied in tertiary hospital settings over a four-year period by Dionisio et al*.* [7] and a 10-year period by Lungren et al. [9], with 19 patients and 111 patients, respectively. 

Our study’s findings are consistent with and reinforce the international literature and medical guidelines on sialorrhea BTX-A injection treatment [11].

Indeed, when relying solely on anatomical landmarks for infiltration, there is a significant risk of missing the target, as illustrated by a study conducted by Loens and colleagues in 2021 [12], which reported a margin of error of approximately 20 mm in locating the glands. Non-radiologist physicians are increasingly using bedside point-of-care ultrasound (POCUS) techniques, and PM&R is not immune to this growing technical evolution, particularly in the treatment of adult musculoskeletal pathologies.

In a recent review, POCUS-guided BTX-A application in salivary glands was highlighted as a technique suggested by most experts for the treatment of anterior sialorrhea in the pediatric population [13]. 

While several studies demonstrate the utility of POCUS, others show how simple it is to achieve an adequate level of structure identification, even by a nonexpert user [14].

As long as there are sufficient resources and tutoring support, the learning curve is short given the low difficulty of the locoregional ultrasound study of the area to be treated.

Concerning the drug's pharmacokinetics, we observed a peak of effectiveness at three months after US-guided application only, which is consistent with previous research [15].

This study is constrained by inherent selection bias because of retrospective data collection. A significant limitation is our small sample size, likely influenced by several factors: restrictions on BTX-A use for sialorrhea stage ≥3, inconsistent availability of anesthesiologists leading to a substantial patient pool requiring treatment, and the intraglandular application of BTX-A by specialists outside of PM&R within the same hospital, of which we lack awareness of the outcomes.

## Conclusions

The study concluded that there are significantly better outcomes up to the third month after sialorrhea BTX-A treatment when performed under US guidance. Our study is relevant as it is, to the authors’ knowledge, the first to address the importance of ultrasound-guided BTX-A injection by POCUS and to demonstrate the success of this application by a physiatrist in a PM&R pediatric department in Portugal. Further research with a larger sample size and prospective design would be beneficial to validate the findings and address the study's limitations.
